# Differential Regulation of Damage-Associated Molecular Pattern Release in a Mouse Model of Skeletal Muscle Ischemia/Reperfusion Injury

**DOI:** 10.3389/fimmu.2021.628822

**Published:** 2021-07-26

**Authors:** Hiroaki Furubeppu, Takashi Ito, Midori Kakuuchi, Tomotsugu Yasuda, Chinatsu Kamikokuryo, Shingo Yamada, Ikuro Maruyama, Yasuyuki Kakihana

**Affiliations:** ^1^ Department of Emergency and Intensive Care Medicine, Kagoshima University Graduate School of Medical and Dental Sciences, Kagoshima, Japan; ^2^ Department of Systems Biology in Thromboregulation, Kagoshima University Graduate School of Medical and Dental Sciences, Kagoshima, Japan; ^3^ R&D Center, Shino-Test Corporation, Sagamihara, Japan

**Keywords:** ischemia reperfusion injury, high mobility group box 1, extracellular histones, damage-associated molecular patterns, skeletal muscle

## Abstract

**Background:**

Skeletal muscle ischemia/reperfusion (I/R) injury is an important clinical issue that can cause remote organ injury. Although its pathogenesis has not been fully elucidated, recent studies have suggested that damage-associated molecular patterns (DAMPs) are mediators of remote organ injury in sterile inflammation. The purpose of this study was to investigate the possible involvement of DAMPs, including the nuclear proteins high-mobility group box 1 (HMGB1) and histone H3, in the pathogenesis of skeletal muscle I/R injury in mice.

**Methods:**

Hindlimb ischemia was induced in mice through bilateral ligation of inguinal regions using rubber grommets. Reperfusion was induced by cutting the rubber grommets after 2–12 h of ischemic period. Survival rates, localization of HMGB1 and histone H3 in the gastrocnemius muscle, and circulating HMGB1 and histone H3 levels were analyzed. The effect of anti-HMGB1 and anti-histone H3 antibodies on survival was analyzed in mice with I/R injury.

**Results:**

All mice with hindlimb ischemia survived for at least 36 h, while all mice died within 24 h if the hindlimbs were reperfused after ischemia for 4–12 h. Immunohistochemical analysis revealed that HMGB1 translocated from the nucleus to the cytoplasm in the ischemic gastrocnemius muscle, while histone H3 was confined to the nucleus. Accordingly, serum HMGB1 levels were significantly elevated in mice with hindlimb I/R compared with normal mice or mice with hindlimb ischemia (*P* < 0.05). Serum histone H3 levels were not elevated after I/R. Treatment with anti-HMGB1 antibodies significantly improved survival of mice with hindlimb I/R injury compared with control antibodies (*P* < 0.05).

**Conclusions:**

HMGB1, but not histone H3, translocated to the cytoplasm during skeletal muscle ischemia, and was released into the systemic circulation after reperfusion in mice with I/R injury. Treatment with anti-HMGB1 antibodies partially improved survival.

## Background

Skeletal muscle ischemia/reperfusion (I/R) injury is a clinical event that is associated with trauma, acute peripheral vascular disease or iatrogenic vascular injury. Crush syndrome is a specific subtype of skeletal muscle I/R injury induced by prolonged, physical compression on the limbs and subsequent release of pressure (1). Reperfusion of ischemic skeletal muscles leads to the release of intracellular components, such as myoglobin, potassium, and damage-associated molecular patterns (DAMPs), to the extracellular space. The release of these molecules induces capillary damage and movement of body fluids into the extravascular space, which causes hypovolemic shock, thrombosis, multiple organ dysfunction, and death (2, 3).

High-mobility group box 1 protein (HMGB1) is a prototypical DAMP molecule. HMGB1 is mainly located in the nucleus where it binds DNA and regulates gene expression. HMGB1 is passively released from damaged cells following sterile tissue injury due to I/R (4). The extracellular HMGB1, as a DAMP molecule, promotes response to sterile injury by stimulating innate immune cells and induces systemic inflammatory response syndrome (5). Histones are the chief protein components of the chromatin and act as spools around which DNA winds. Histones are released from damaged cells or neutrophils by means of neutrophil extracellular traps. The extracellular histones promote inflammation, tissue injury and organ damage, in part through binding specifically to Toll-like receptors and non-specifically to plasma membrane (6).

Previous studies have suggested possible involvement of HMGB1 and histones in the pathogenesis of I/R injury in various organs (6, 7). However, detailed behavior, such as spatiotemporal dynamics of multiple DAMP molecules in I/R injury, remains undetermined. Here, using a mouse model of skeletal muscle I/R injury, we show that HMGB1, but not histone H3, translocated from the nucleus to the cytoplasm during skeletal muscle ischemia, and was released into the systemic circulation immediately after reperfusion.

## Methods

### Animals

All experimental procedures were performed in accordance with the guiding principles for the care and use of animals in the field of physiological sciences published by the Physiological Society of Japan (2015), and approved by the Institutional Animal Use Committees at Kagoshima University (MD14058, MD16018, MD16113, MD18123 and MD20096). C57BL/6 male mice (CLEA JAPAN, Inc., Tokyo, Japan) were housed at room temperature (25–26°C) with a 12-h light/dark cycle and allowed free access to standard pellet chow and water. All mice were anesthetized with isoflurane during the I/R procedure and drug administration. At all other times, the mice had free access to food and water.

### Hindlimb I/R

Hindlimb ischemia was achieved at the age of 8 to 12 weeks by putting a rubber grommet (5 mm, KGE-5A, HIKARI Co., Ltd., Osaka, Japan) on their groin, and reperfusion was initiated by cutting the grommet. Ischemia and reperfusion of the limbs were confirmed by the change in the color and size of the paws. The mice were sacrificed by anesthetic overdose. Blood samples were then collected, and the gastrocnemius muscle was harvested for histology.

### Survival Rate

Mice underwent ischemia without reperfusion or 2–12 h ischemia followed by reperfusion. The general condition of each mouse was evaluated every 1 h during the reperfusion phase. Mice were sacrificed when moribund or 24 h after reperfusion, and their survival rates after reperfusion were investigated. In another experiment, mice with 3–12 h ischemia followed by reperfusion were randomly allocated into any of the following groups: those injected intraperitoneally with mouse anti-HMGB1 IgG (Shino-Test Corporation, Sagamihara, Japan); those injected intraperitoneally with chicken anti-histone H3 IgY (Shino-Test Corporation); those injected intraperitoneally with both anti-HMGB1 IgG and anti-histone H3 IgY; those injected intraperitoneally with control IgG and/or IgY (Shino-Test Corporation).

### Measurement of Serum Creatine Kinase, HMGB1, Histone H3, and Tumor Necrosis Factor-α Levels

Blood and tissue samples were collected from euthanized mice with or without I/R. Blood samples were centrifuged at 2000 × *g* for 10 min to separate the serum. Serum HMGB1, acetylated HMGB1, histone H3, and citrullinated histone H3 levels were measured using an enzyme-linked immunosorbent assay (ELISA) at Shino-Test Corporation as described previously (8). Serum CK levels were analyzed using SPOTCHEM EZ (SP-4430, ARKRAY, Kyoto, Japan). Serum TNF-α levels were measured using a mouse TNF-α ELISA kit (R&D systems, Minneapolis, MN, USA).

### Histological Examinations

The harvested gastrocnemius muscles, lungs, and kidneys were preserved in 4% paraformaldehyde (Fujifilm Wako Pure Chemical Corporation, Osaka, Japan) and embedded in paraffin. Sections were stained with hematoxylin and eosin (H&E). For immunohistochemical analysis, muscle sections were blocked with Block ACE (Dainippon Sumitomo Parma Co, Tokyo, Japan) and incubated with rabbit anti-HMGB1 antibody (Abcam, Cambridge, UK), rabbit anti-histone H3 antibody (Abcam), or normal rabbit IgG (Merck KGaA, Darmstadt, Germany) at 4°C overnight. The primary antibodies were detected using peroxidase-conjugated goat anti-rabbit IgG antibody (N-Histofine^®^ Simple Stain MAX PO, Nichirei Biosciences Inc., Tokyo, Japan). Sections were observed under a light microscope (BZ-X710, KEYENCE Co., Osaka, Japan). In each section, the ratios of HMGB1-positive and histone H3-positive nuclei were calculated in 10 high-power fields.

## Statistics

Statistical analysis was performed using SPSS version 24.0 (SPSS Inc., Chicago, IL, USA). Data are shown as box plots with lower extreme, lower quartile, median, upper quartile and upper extreme values. Data were tested for normal distribution using the Shapiro–Wilk test. Normally distributed data were compared using Tukey’s honest significance test or the Games–Howell test. Data with non-normal distribution were compared using the Kruskal–Wallis test. Survival curves were calculated using the Kaplan–Meier method. Survival rates were compared by a log-rank test. Differences were considered significant at *P* < 0.05.

## Results

### Survival Rate

**Figure 1:**
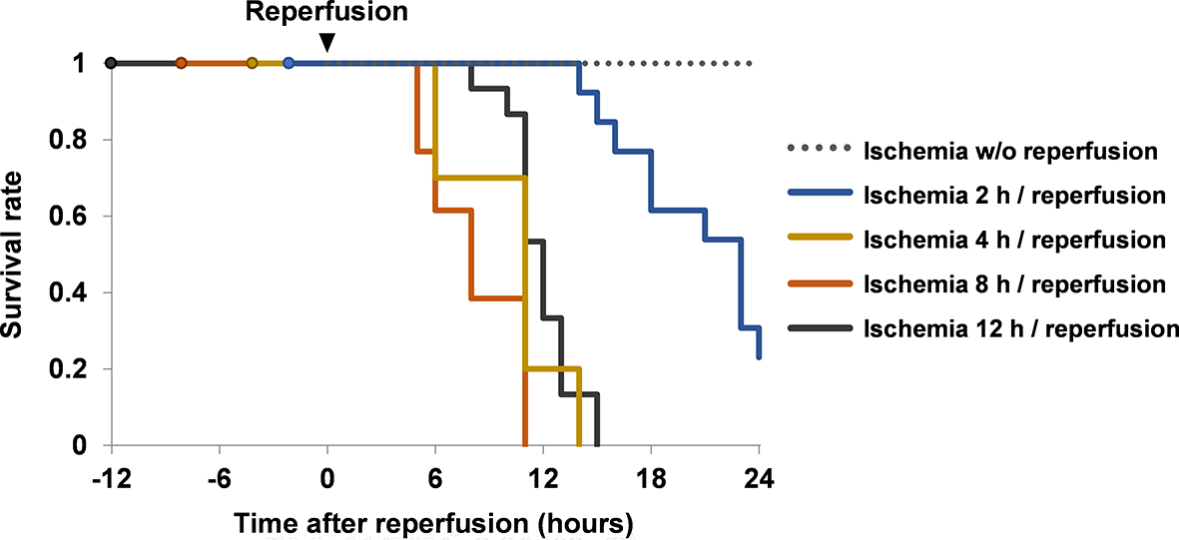
Survival rates of mice with ischemia and reperfusion. Mice were divided into ischemia without reperfusion group (*n* = 8), ischemia 2 h/reperfusion group (*n* = 13), ischemia 4 h/reperfusion group (*n* = 10), ischemia 8 h/reperfusion group (*n* = 13) and ischemia 12 h/reperfusion group (*n* = 15). Their survival rates were investigated up to 24 h after reperfusion. Cumulative data of two or more independent experiments are shown. w/o, without.

Mice with hindlimb ischemia survived for at least 36 h if the hindlimbs were kept constricted (**Figure 1**). Conversely, all mice died within 24 h if the hindlimbs were liberated and reperfused after ischemia for 4–12 h.

### Serum Levels of HMGB1, Histone H3, and CK

**Figure 2:**
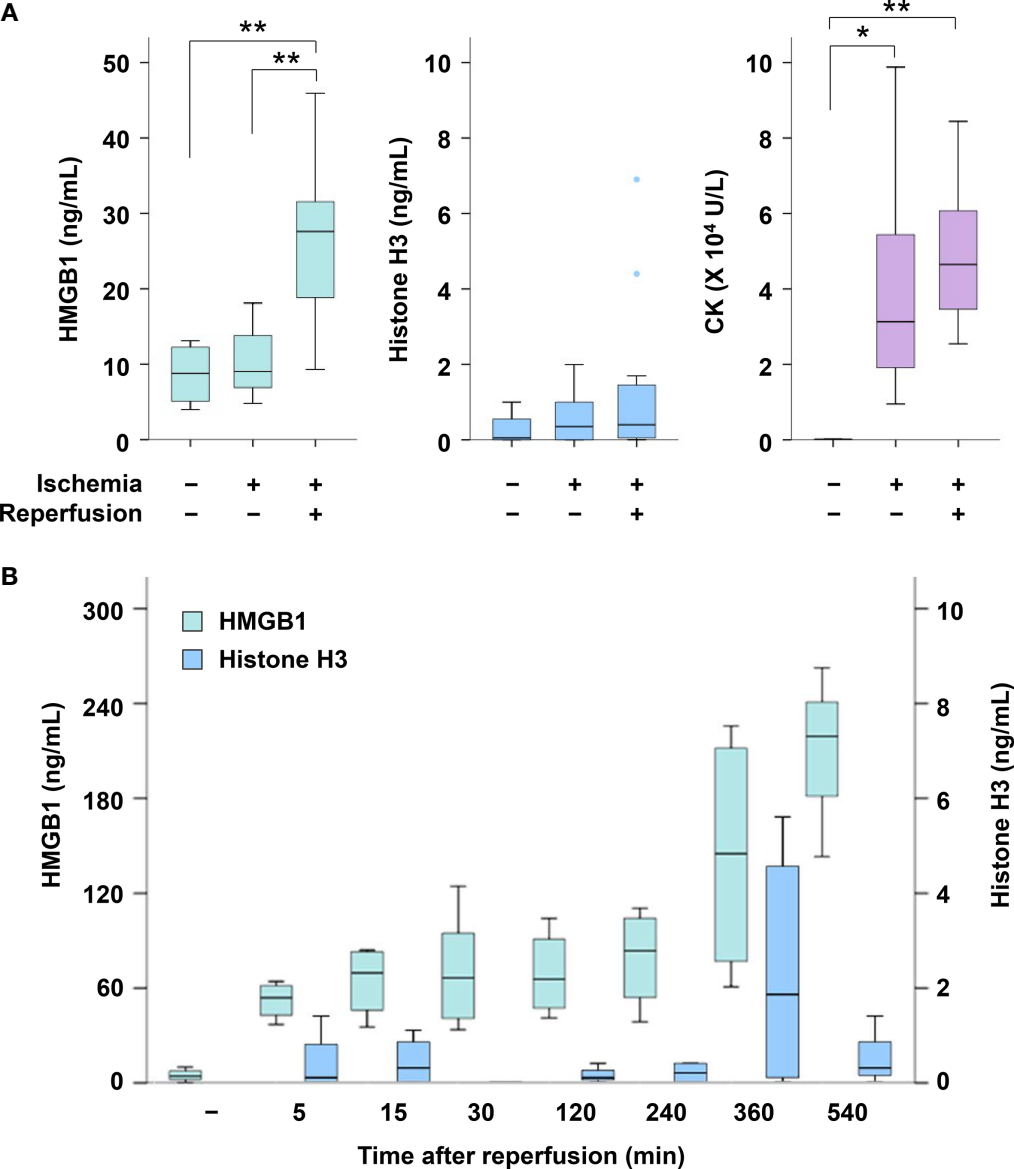
Serum levels of HMGB1, histone H3 and CK in mice with or without I/R. **(A)** Serum levels of HMGB1, histone H3 and CK were measured in normal mice (*n* = 4), mice with 12 h of ischemia (*n* = 8), and mice with 12 h of ischemia followed by 30 min of reperfusion (*n* = 15). *P < 0.05, **P < 0.01. **(B)** Serum levels of HMGB1 and histone H3 were measured at 5 min (*n* = 4), 15 min (*n* = 4), 30 min (*n* = 6), 120 min (*n* = 4), 240 min (*n* = 4), 360 min (*n* = 4) and 540 min (*n* = 3) after I/R. Representative data of two independent experiments are shown.

The serum levels of CK were increased in mice with hindlimb ischemia compared with normal mice (*P* < 0.05, **Figure 2A**). The serum levels of HMGB1 were not increased during 12 h ischemia; however, they were significantly elevated after reperfusion (*P* < 0.05, 12 h ischemia without reperfusion vs. 12 h I/R). They were elevated soon after reperfusion, and remained elevated for several hours (**Figure 2B**). The serum HMGB1 levels were also increased after 2 h I/R, albeit to a lesser extent (**Additional Figure 1A**). Neither histone H3 nor citrullinated histone H3 was elevated in mice with ischemia or I/R (**Figure 2A** and **Additional Figure 1B**).

### Translocation of HMGB1 After Ischemia and I/R in the Gastrocnemius Muscle

**Figure 3:**
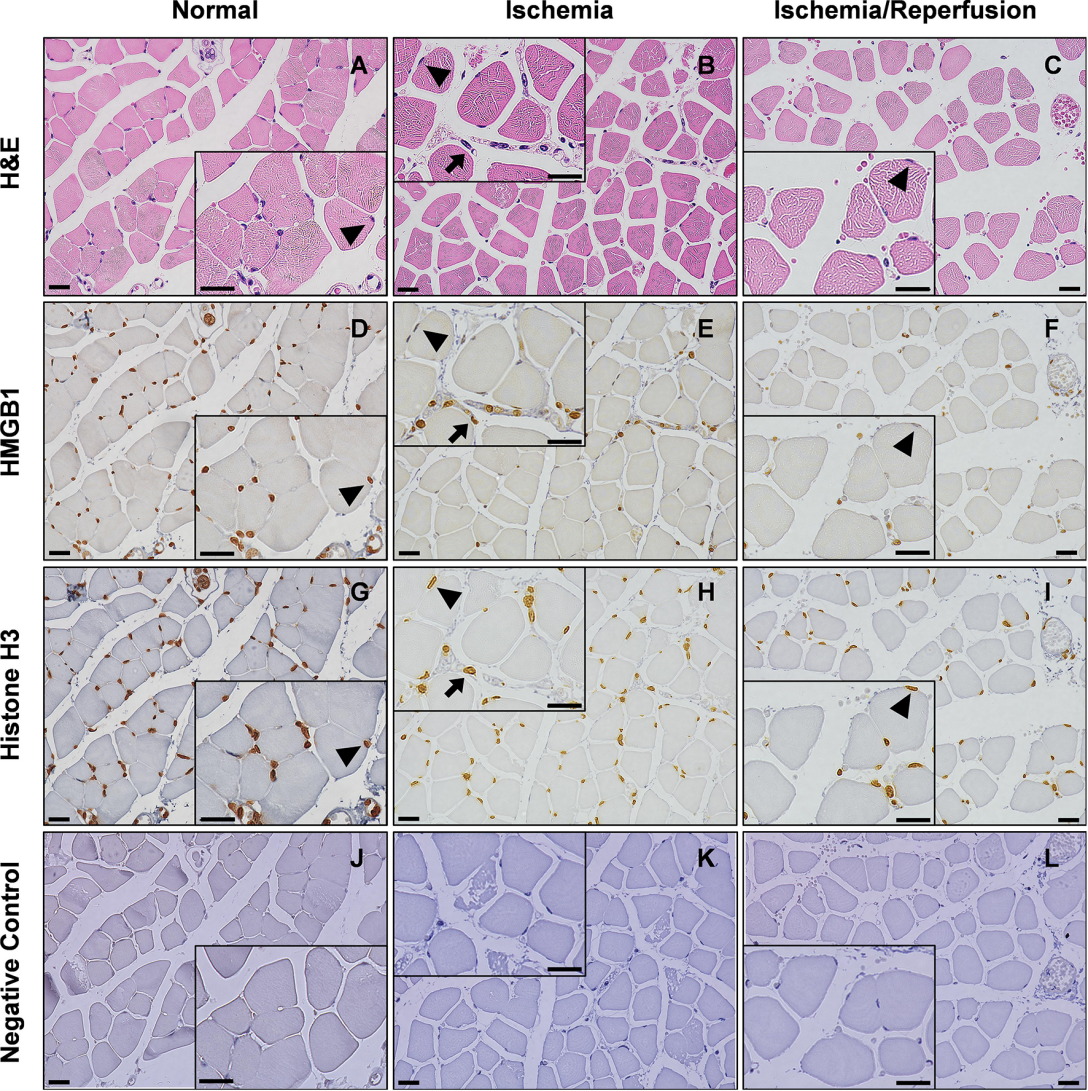
Translocation of HMGB1 after I/R in mice. Light microscopy view of the hindlimb muscle. Serial sections of gastrocnemius muscles were stained with H&E **(A–C)**, anti-HMGB1 antibodies **(D–F)**, anti-histone H3 antibodies **(G–I)**, or non-immune negative control **(J–L)** in normal, 12 h ischemia, and 12 h I/R mice. Insets show magnified images of skeletal muscle cells and leukocytes. Arrow heads indicate that HMGB1 and histone H3 are abundantly expressed in the nucleus of normal skeletal muscle cells while HMGB1, but not histone H3, disappears from the nucleus after ischemia or I/R. Arrows indicate that HMGB1 and histone H3 are abundantly expressed in the nucleus of leukocytes even after ischemia. The scale bars indicate 20 μm. Representative images of two or more independent experiments are shown.

As shown in **Figure 3**, degeneration and swelling of muscle fibers and infiltration of leukocytes were observed after ischemia and I/R. Immunohistochemical analysis revealed that HMGB1 staining was localized in the nucleus in normal muscles, while nuclear HMGB1 disappeared after ischemia (**Figures 3E, F**, **Additional Figure 2A**). Cytoplasmic HMGB1 slightly increased during ischemia and decreased after reperfusion. In contrast, histone H3 staining was mostly confined to the nucleus even after ischemia and I/R (**Figures 3H, I**, **Additional Figure 2B**).

### Effects of Anti-HMGB1 and/or Anti-Histone H3 Antibodies on Hindlimb I/R Injury

**Figure 4:**
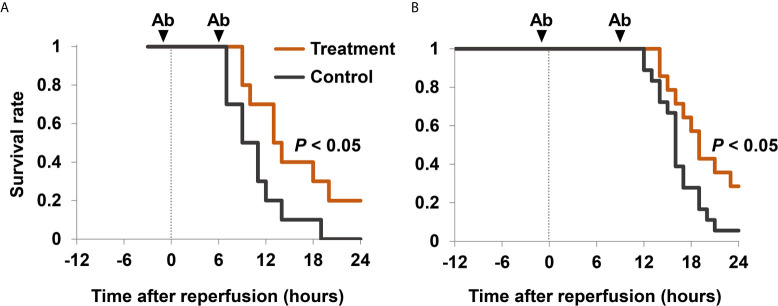
Survival rates after I/R in mice treated with anti-HMGB1 and anti-histone H3 antibodies. **(A)** Mice with 3 h I/R injury were treated with 2.5 mg/kg of anti-HMGB1 antibodies (*n* = 10) or non-specific control antibodies (*n* = 10) twice, at 1 h before reperfusion and 6 h after reperfusion. **(B)** Mice with 12 h I/R injury were treated with 2.5 mg/kg of anti-HMGB1 and anti-histone H3 antibodies (*n* = 14) or non-specific control antibodies (*n* = 18) twice, at 1 h before and 9 h after reperfusion. Mice also received a subcutaneous injection of 20 mL/kg of normal saline 1 h before reperfusion. Survival curves were calculated using the Kaplan–Meier method. Survival rates were compared using a log-rank test. Cumulative data of two independent experiments are shown. Ab, antibodies.

To examine whether extracellular HMGB1 has pathogenic roles in hindlimb I/R injury, we analyzed the survival rates of mice in the presence or absence of HMGB1 neutralizing antibodies. Treatment with anti-HMGB1 antibodies significantly improved survival in mice with 3 h I/R (**Figure 4A**) but not in mice with 4 h I/R (**Additional Figure 3A**) or 12 h I/R (data not shown). The concurrent use of anti-HMGB1 antibodies and anti-histone H3 antibodies significantly improved the survival rate after 12 h I/R (**Figure 4B**). Although these antibodies did not decrease serum levels of proinflammatory cytokine TNF-α (**Additional Figure 3B**), they reduced lung inflammation and kidney injury in mice with 12 h I/R (**Additional Figure 4A**).

## Discussion

The results of this study demonstrated the following main findings. First, translocation of HMGB1 from the nucleus to the cytoplasm was observed during the ischemia phase, whereas this was not observed with histone H3. Second, HMGB1, but not histone H3, was released to the systemic circulation after reperfusion. Third, anti-HMGB1 antibodies partially improved survival after I/R injury.

In our study, histology was used to test the reliability of our I/R injury model. There was swelling and degeneration of the gastrocnemius muscle, which indicated successful establishment of the I/R injury model. The 12-h period of ischemia was selected for the severe I/R injury model because the mean time under the rubble of crush syndrome patients is 11.7 ± 14.3 h (9).

It has been reported that crush syndrome can lead to fatal arrhythmia associated with hyperkalemia, shock, acute renal failure, multiple organ failure and finally death, and that the injury of skeletal muscle cells occurs mainly during the reperfusion phase rather than ischemia phase (1, 3). Reperfusion in ischemic muscles induces the release of myoglobin, the generation of oxygen free radicals, and the formation of obstructive thrombi within the vasculature (3). Our present study supports this phenomenon, because all mice in the ischemia group remained in good condition in the upper body and survived for at least 36 h, whereas All mice with hindlimb ischemia survived for at least 36 h, while all mice died within 24 h if the hindlimbs were reperfused after ischemia for 4–12 h. Thus, the general condition of mice was exacerbated after reperfusion and this was accompanied by the elevation of serum HMGB1 levels.

HMGB1 is an important mediator of I/R injury in the liver, heart, kidney, spinal cord, brain and intestine, and of crush injury (4, 7, 9, 10). It has been reported that serum levels of HMGB1 were elevated within 30 min after severe trauma (11) and at 3 h after crush injury (7). In the present study, serum levels of HMGB1 were elevated soon after reperfusion, and remained elevated for several hours. Although serum HMGB1 levels were not increased during the ischemic phase, histological analysis revealed that most of the HMGB1 translocated from the nucleus to the cytoplasm in this phase. Subsequently, reperfusion may stimulate HMGB1 release into the extracellular space. Translocation of HMGB1 from the nucleus to the cytoplasm was also confirmed in cultured skeletal muscle cells (data not shown), although *in vitro* experiments represented hypoxic conditions rather than I/R. Treatment with anti-HMGB1 antibodies significantly improved survival in mice with 3 h I/R but not in mice with 4 h I/R or 12 h I/R, suggesting the window of opportunity for the treatment in this I/R model.

The serum levels of histones were elevated in animal models of I/R injury in the liver, kidney, lung and brain (6). It has been reported that the serum levels of histones in critically injured trauma patients were elevated on admission (12) and the serum levels of histones in a severe trauma animal model were elevated 1 h after trauma (13). However, the serum levels of histone H3 in our I/R model showed no increase. Our findings indicated that nuclear HMGB1 easily translocated to the cytoplasm and subsequently to the serum in skeletal muscle I/R injury, whereas histone H3 did not. This is possibly because HMGB1 binds to chromatin more loosely than histone H3 (14) and thus can be more easily released to the extracellular space (15) during skeletal muscle I/R injury. It is also possible that intracellular HMGB1 inhibits inflammatory nucleosome (histones and DNA) release (16). More extensive destruction of tissues may therefore be required for the extracellular release of histones. In this context, histones might be released extracellularly in the local compression site, which was not available for histological analysis in this study because of severe tissue destruction, and might contribute to deterioration of the prognosis, even though circulating histone H3 did not increase.

There were several limitations in this study. First, our results obtained from mice cannot be directly applied to I/R injury in clinical practice. However, because there are almost no species differences in the amino acid sequences of HMGB1 and histone H3, the measurement system and neutralizing antibodies can be used in both species. Second, the cause of death in our I/R injury model is controversial. DAMPs are partially attributable because antibodies against them partially improved the survival after I/R. DAMPs released from damaged muscles might contribute to the remote organ injury, such as acute lung injury and acute kidney injury. Factors other than DAMPs, including myoglobin and oxygen free radicals, might also be important (17). Hypovolemia might also be involved because decreased circulating blood volume was noted despite the subcutaneous fluid replacement of 20 mL/kg. Fatal arrhythmia associated with hyperkalemia is less likely to be involved because it commonly occurs immediately after reperfusion, which are contrary to the death seen in our I/R model in half a day. Further studies are needed to understand how DAMPs and other factors contribute to the death in I/R injury.

## Conclusions

HMGB1, but not histone H3, translocated from the nucleus to the cytoplasm during skeletal muscle ischemia and was released to the systemic circulation immediately after reperfusion in mice with I/R injury. Treatment with anti-HMGB1 antibodies partially improved the survival of mice with skeletal muscle I/R injury.

## Data Availability Statement

The raw data supporting the conclusions of this article will be made available by the authors, without undue reservation.

## Ethics Statement

The animal study was reviewed and approved by Institutional Animal Use Committees at Kagoshima University.

## Author Contributions

HF and TI designed the experimental protocol and wrote the manuscript. HF and CK performed blood analysis of mice and the immunohistochemical analysis of the gastrocnemius muscle. HF and MK performed the immunofluorescence analysis of the cell cultures. HF, TI, CK, SY, and TY analyzed laboratory data. IM and YK critically appraised the manuscript. All authors contributed to the article and approved the submitted version.

## Funding

This work was supported by a research grant from the Japan Society for the Promotion of Science (17K17056).

## Conflict of Interest

The ELISAs for HMGB1, acetylated HMGB1, histone H3, and citrullinated histone H3 are manufactured by Shino-Test Corporation, where SY is an employee. TI and IM hold endowed faculty positions at Kagoshima University and receives research funding from Shino-Test Corporation. The funding is for academic promotion and is not directly related to the present study.

The remaining authors declare that the research was conducted in the absence of any commercial or financial relationships that could be construed as a potential conflict of interest.

## Publisher’s Note

All claims expressed in this article are solely those of the authors and do not necessarily represent those of their affiliated organizations, or those of the publisher, the editors and the reviewers. Any product that may be evaluated in this article, or claim that may be made by its manufacturer, is not guaranteed or endorsed by the publisher.

## Abbreviations

CK, creatine kinase; DAMPs, damage-associated molecular patterns; ELISA, enzyme-linked immunosorbent assay; H&E, hematoxylin and eosin; HMGB1, high-mobility group box 1 protein; I/R, ischemia/reperfusion.
