# Supplementary material for: Differential Regulation of Damage-Associated Molecular Pattern Release in a Mouse Model of Skeletal Muscle Ischemia/Reperfusion Injury
Source: Front Immunol. 2021 Jul 26;12:628822. doi: 10.3389/fimmu.2021.628822 (PMC8350322; doi:10.3389/fimmu.2021.628822)
Supplement: Supplementary file 1 [file DataSheet_1.pdf]

Additional Figure 1

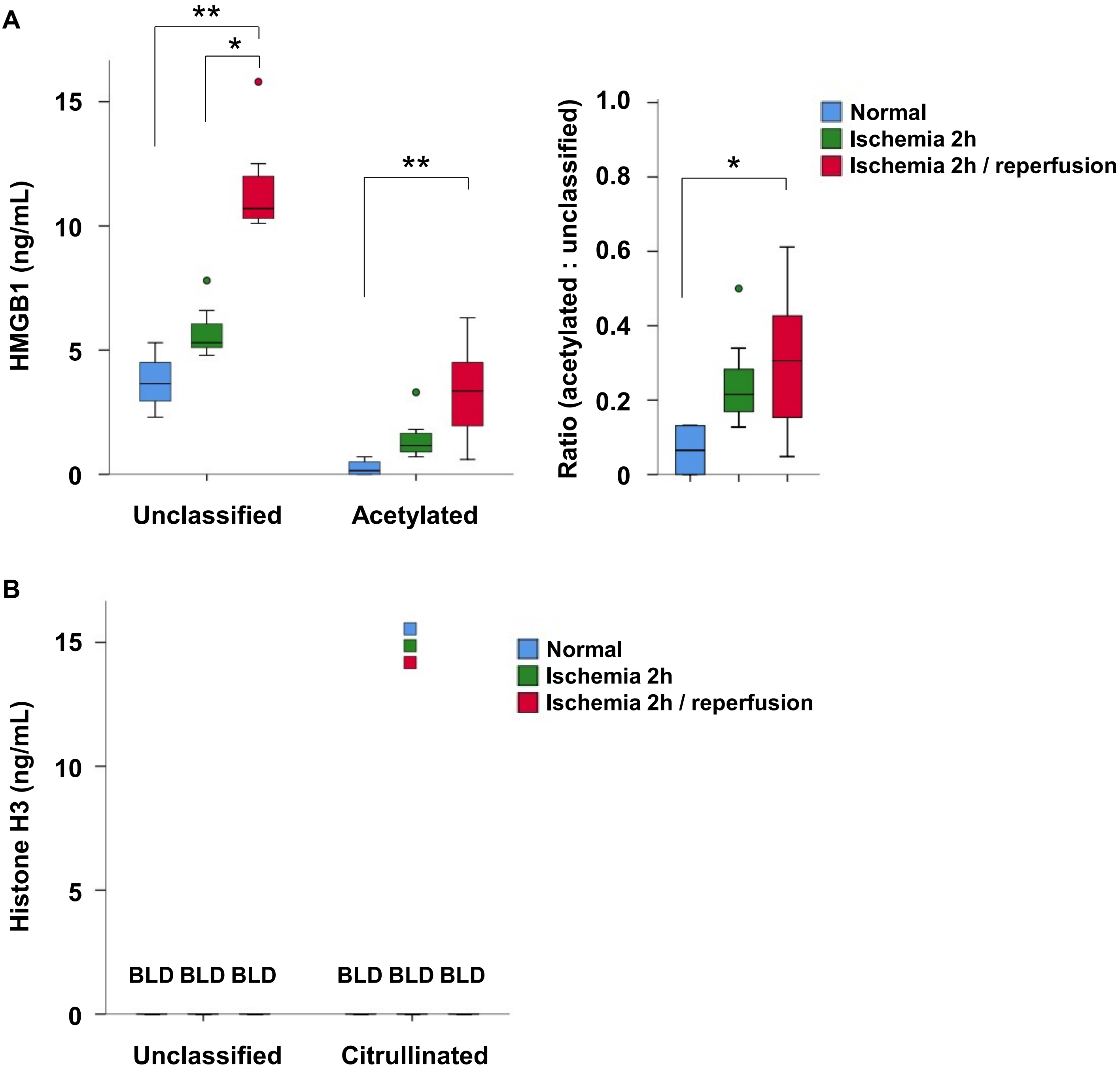

Additional Figure 1. Serum levels of HMGB1 and histone H3 with or without 2 h ischemia and reperfusion.

(A) Serum levels of HMGB1, acetylated HMGB1, and the ratio of acetylated HMGB1 to unclassified HMGB1 were measured in normal mice (n = 4), mice with 2 h ischemia (n = 8), and mice with 2 h I/R (n = 8). The ratio of acetylated HMGB1 was increased in I/R mice, suggesting that active secretion, rather than passive release, of HMGB1 might be involved in the elevation of serum HMGB1 levels in I/R mice. **P* < 0.05. ***P* < 0.01. (B) Serum levels of histone H3 and citrullinated histone H3 were measured in normal mice (n = 4), mice with 2 h ischemia (n = 8), and mice with 2 h I/R (n = 8). BLD: below the limit of detection.

Additional Figure 2

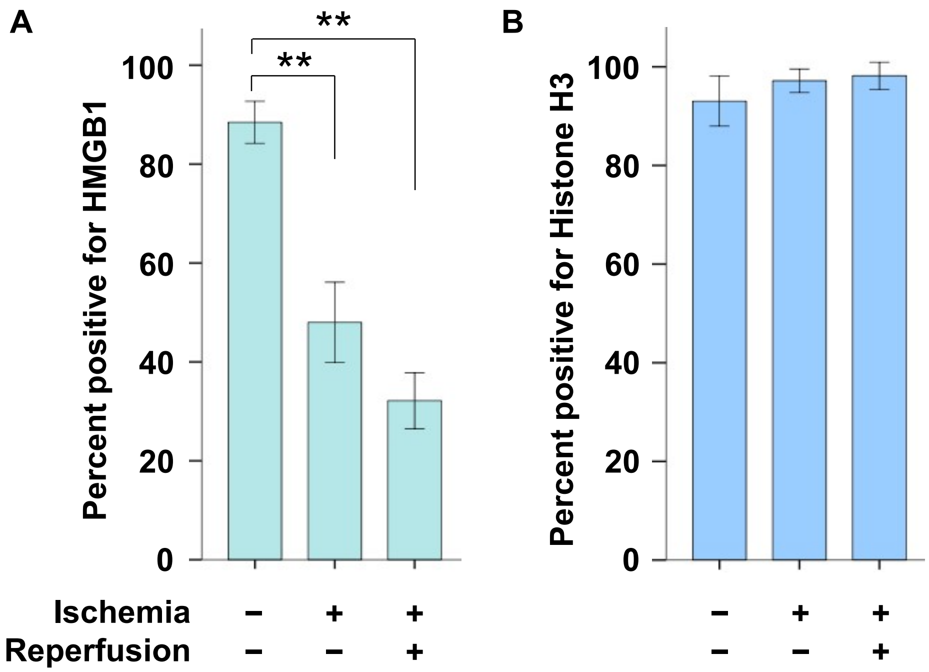

Additional Figure 2. Nuclear HMGB1 disappears after ischemia, related to Figure 3.
The percentages of HMGB1-positive and histone H3-positive nuclei were calculated in 30 high-power fields from three muscle sections in each group (normal, 12 h ischemia, and 12 h I/R). Data are shown as mean ± SD. ***P* < 0.01.

Additional Figure 3

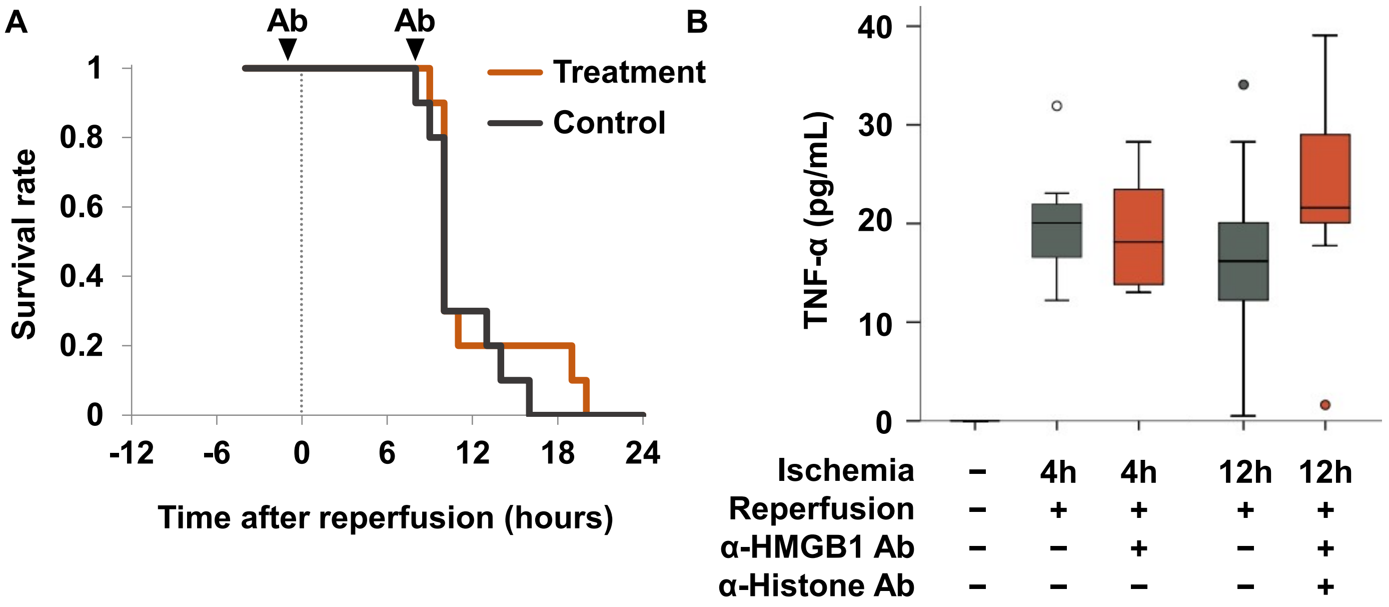

Additional Figure 3. Effects of anti-HMGB1 antibodies against I/R injury in mice.
(A) Mice with 4 h hindlimb ischemia followed by reperfusion were treated with 2.5 mg/kg of anti-HMGB1 antibodies (n = 10, orange line) or non-specific control antibodies (n = 10, gray line) twice at 1 h before reperfusion and 8 h after reperfusion. Survival rates were investigated up to 24 h after reperfusion and compared using a log-rank test. (B) Serum levels of TNF-α were measured in mice with 4 h or 12 h hindlimb ischemia and reperfusion treated with anti-HMGB1 antibodies, anti-histone H3 antibodies, or non-specific control antibodies. There were no statistically significant differences between the treatment group and the control group. Abbreviations: Ab, antibodies.

Additional Figure 4

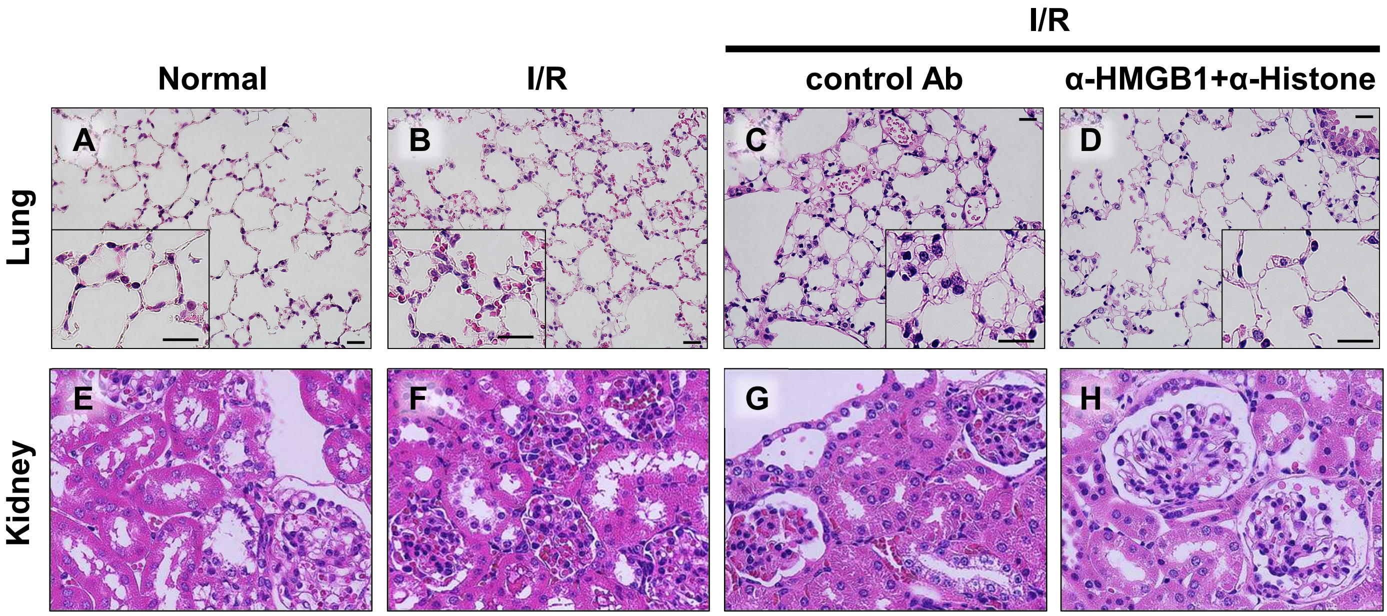

Additional Figure 4. Anti-HMGB1 and anti-histone H3 antibodies suppress remote organ injury in mice with ischemia/reperfusion (I/R).

(A–D) Lungs in normal mice (A), I/R mice (B), I/R mice treated with control antibodies (C), and I/R mice treated with anti-HMGB1 and anti-histone H3 antibodies (D) were stained with H&E. Insets show magnified images of alveolar capillaries. Anti-HMGB1 and anti-histone H3 antibodies suppressed leukocyte accumulation in the lung in mice with I/R. The scale bars indicate 20 μm. (E–H) Kidneys in normal mice (E), I/R mice (F), I/R mice treated with control antibodies (G), and I/R mice treated with anti-HMGB1 and anti-histone H3 antibodies (H) were stained with H&E. Anti-HMGB1 and anti-histone H3 antibodies suppressed renal tubular degeneration and glomerular damage in mice with I/R.
